# Successful self-employment in microenterprise for persons with disabilities in a rural setting

**DOI:** 10.4102/ajod.v14i0.1564

**Published:** 2025-02-28

**Authors:** Luther L. Monareng, Mogammad S. Soeker, Deshini Naidoo

**Affiliations:** 1 Department of Occupational Therapy, College of Health Sciences, University of KwaZulu-Natal, Durban, South Africa; 2 Department of Occupational Therapy, College of Health Sciences, University of Western Cape, Bellville, South Africa

**Keywords:** employment, entrepreneurship, income-generation, microenterprise, therapy, vocational rehabilitation, work

## Abstract

**Background:**

Persons with disabilities are involved in self-employment (vocational rehabilitation) in microenterprises despite key role players not making valuable contributions or using self-employment as a placement option.

**Objectives:**

This research aimed to explore profitable self-employment microenterprises for persons with disabilities in rural South Africa.

**Method:**

This qualitative research study was conducted in a rural community in KwaZulu-Natal province, South Africa. Purposive and snowball sampling were used to recruit 10 persons with disabilities running profitable microenterprises for an average of 5 years each. Data were collected using a piloted question guide in a face-to-face interview. Thematic analysis followed the hybrid inductive and deductive approaches.

**Results:**

Persons with disabilities participated, and 9 out of 10 were males. Two themes emerged. Theme one: Running microenterprises – self-initiated and maintained. They use their hands and minds to start microenterprises that benefit them and their families. Theme two: Multiple key role players should be involved in self-employment. Persons with disabilities perceive various key role players and themselves as having an active role in self-employment to benefit their microenterprises.

**Conclusion:**

Persons with disabilities in a rural setting engage in successful self-employment in microenterprises, which they self-initiate and maintain. Roles and responsibilities of persons with disabilities and key role players in and outside the hospital setting are crucial for those in self-employment.

**Contribution:**

This research generated contextual data towards the under-researched subject on self-employment for persons with disabilities.

## Introduction

In developing countries, such as South Africa, unemployment for persons with disabilities is generally high despite national and international bodies calling for their inclusion in employment (Department of Labour 1998; Morwane, Dada & Bornman 2021). South Africa’s soaring unemployment rate is 31.9% (Statistics South Africa 2023). The highest unemployment rate among the country’s nine provinces is in the Eastern Cape (38.5%) followed by KwaZulu-Natal (29.4%), while the lowest is in the Western Cape (20.2%) (STATSSA 2023). Persons with disabilities make up 80% of the working-age population and face unemployment rates that are approximately twice those of their non-disabled counterparts (Blanck et al. 1999; International Labour Organization 2019; Quinton 2014; World Health Organization & World Bank 2011; Yamamoto & Alverson 2015). Furthermore, amid these alarming unemployment statistics, employment in developing countries is generally sought in the informal sector, as evident in South Africa (STATSSA 2019, 2023). Mahadea and Khumalo (2020) reported that microenterprises (small businesses) make up 75% of businesses globally, contributing about 60% towards job creation or employment.

Fanta et al. (2017) added that the limited support from relevant key role players contributes to the high failure rates for those self-employed in microenterprises in developing countries. In addition, despite the lack of support, those who are self-employed in microenterprises, including persons with disabilities, prefer to stay in the informal sector as there are limited barriers to entry (Mahadea & Khumalo 2020). In contrast, there are high costs associated with the formal sector, such as tax and regulations (Mahadea & Khumalo 2020). Furthermore, Isaacs et al. (2007) stated that the benefits of self-employment entail that jobs are created in a country to combat unemployment, contribute to economic growth and improve living standards.

Isaacs et al. (2007) alluded to the importance of starting training at school level, as observed in industrialised countries, to enhance a country’s efforts in self-employment. Self-employment or entrepreneurship training increases the chances of succeeding in business as an individual will have the knowledge and skills, that is the know-how to run a business (Chimucheka 2014). Unlike in the past, modern-day entrepreneurship is taught to anyone willing to be educated, irrespective of their level of education (Alberti, Sciascia & Poli 2004; Brockhaus 2001). According to Ladzani and Van Vuuren (2002), beyond training an individual on being entrepreneurial (such as being creative and identifying opportunities) and on business skills (such as managing the business and finances), they deemed motivation to be of significance as it contributes to an individual being able to overcome obstacles, remain inspired and have the ability to seek assistance when necessary. In contrast, Wasike and Likoko (2013) support customised training in entrepreneurship for persons with disabilities to accommodate their respective needs and ensure equality. Furthermore, the Hytti et al. (2002) model promotes entrepreneurship education, highlighting crucial aspects such as becoming an entrepreneur and a business manager.

The above-stated information suggests that there is a need for necessary resources to be made available or mobilised to make self-employment a reality, especially for vulnerable groups, such as persons with disabilities. However, research shows that resources are generally limited, if not non-existent, in lower-economy countries (Maseko et al. 2023; World Bank 2022). Shakespeare et al. (2019) added that resources are even less in rural settings compared to urban settings. As a result, although entrepreneurship training programmes are available in developing countries such as South Africa, training institutions in urban settings are likely to be more resourced than those in rural settings. On the availability of resources in self-employment training institutions, Isaacs et al. (2007) reported a challenge in the availability of qualified and trained educators.

Beyond the necessity of training and an individual’s capabilities towards realising self-employment, Binti, Siti and Buntat (2012) perceive the following to be key role players, namely, the government to create a conducive environment such as offering business grants, cutting the bureaucracy by promulgating laws; family to provide necessary support; and persons with disabilities and communities to work in unity to raise awareness in society. Other key role players are therapists who offer services to persons with disabilities, including vocational rehabilitation (Buys 2015; Soeker 2017), at different levels of care, from specialised hospitals to clinics in communities near service users (Van Biljon et al. 2016). However, these professionals are unclear about what specific vocational rehabilitation to offer at each level of care, let alone their role in self-employment (Monareng, Franzsen & Van Biljon 2018; Van Biljon et al. 2016). Furthermore, Chimara, Niekerk and Van Biljon (2022) support overt and transparent self-employment initiatives in low-resourced countries. In addition, Gamieldien and Van Niekerk (2017) call for a collaborative approach and effort by all key role players involved in this field, such as the education sector, social development department, labour department, and trade and industry department.

Although persons with disabilities are involved in self-employment in microenterprises for income generation, key role players do not seem to be making valuable contributions or using self-employment as a placement option (Chimara, Van Niekerk & Van Biljon 2021; Van Biljon, Rabothata & De Wit 2019), especially in rural settings where there is much need as resources are limited. To make a contextual contribution to the collaborative approach, key role players in self-employment need to explore crucial aspects in this field. Aspects such as suitable and successful or profitable microenterprises, how one starts and stays self-employed, and to understand the prominent reason persons with disabilities explore self-employment.

### Research question

The research question that this study aims to answer is: ‘What are the key factors that contribute to successful self-employment microenterprises for persons with disabilities in rural KwaZulu-Natal?’

### Aim

The research study aims to explore successful self-employment in microenterprises for persons with disabilities in a rural setting in South Africa.

### Objectives

The above-stated research question will be answered by fulfilling the following objectives in a rural setting of KwaZulu-Natal, South Africa:

- Explore persons with disabilities’ experiences in initiating and maintaining self-employment in microenterprises and
- Explore persons with disabilities’ perceptions of key role players in self-employment in microenterprises.

## Research methods and design

### Study design

Qualitative approach was used in this exploratively designed study (Kielhofner 2017) to explore successful or profitable self-employment microenterprises for persons with disabilities in rural South Africa.

### Setting

Persons with disabilities who are self-employed in microenterprises in a rural and low-resourced community of Manguzi, KwaZulu-Natal province in South Africa, were interviewed. Manguzi is an appropriate setting as it represents a rural setting found in South Africa, where persons with disabilities served by professionals in vocational rehabilitation live.

### Study population and sampling strategy

A total of 10 participants took part in this research, allowing data saturation, that is when no new data emerged (Hennink, Kaiser & Marconi 2017). Purposive sampling was used, followed by snowball sampling to supplement the sample size (Hennink et al. 2017). None of the invited participants refused to participate in this research. Those included had to meet the following criteria:

- A South African of working age (18–65 years),
- Run a business for ≥ 3 years or make a living from the business earnings, and
- Live with a disability.

### Data collection

A research guide was developed based on literature aligned with this research’s question and objectives. The question guide and probing questions were piloted for reliability and validity. Adjustments made to the question guide post the pilot study predominantly were to rephrase and merge similar questions. All semi-structured interviews were conducted one-on-one and face-to-face using persons with disabilities’ preferred language by a trained interviewer with over 20 years of experience in community work. The audio-recorded interviews, 40 minutes each, were transcribed verbatim.

### Data analysis

Thematic data analysis was done using a hybrid inductive and deductive approach (Fereday & Muir-Cochrane 2006). The NVIVO software was used to manage, organise and code the data (Fereday & Muir-Cochrane 2006). Data were cleaned by reading each transcript while listening to the relevant audio recording. To enhance rigour (Koch 1994), credibility and trustworthiness (Aroni et al. 1999), a hybrid approach of inductive and deductive (Fereday & Muir-Cochrane 2006) methods was taken. The main author interpreted the transcribed data and presented it to the other two authors for transparency and further critique (Fereday & Muir-Cochrane 2006), enhancing the rigour of this research.

### Ethical considerations

Ethical approval to conduct the study was received from the University of KwaZulu Natal Biomedical Research Ethics Committee (BREC) (No. BREC/00004655/2022). All participants received the research information sheet and signed a consent form. Helsinki’s Declaration was upheld throughout this research, including voluntary participation and ensuring the confidentiality of data by using pseudonyms (American Medical Association 2013).

## Results

This section presents the participants’ (persons with disabilities) demographics and the two emerging themes. Theme one: Running microenterprises – self-initiated and maintained. Theme two: Multiple key role players should be involved in self-employment.

### Demographics

The demographics of persons with disabilities who are self-employed in microenterprises are outlined in Table 1. Out of 10 persons with disabilities, nine were male. All were sole owners of their microenterprises and employed others, and they were between the age of 35 and 63 years. Their education levels ranged from no formal education to grade 11. All persons with disabilities had physical conditions. While five of them reported that the disability impacted running their business, four stated that the disability had no impact, and one remained neutral.

**TABLE 1:** Demographics of persons with disabilities in self-employment in microenterprises in a rural setting.

Pseudonyms	Gender	Age (in years)	Sole owner	Employ others	Marital status	Dependants feed	Education	Disability/Condition	Impact of disability
VM	Male	49	Yes	Yes	Single	02	None	Spinal cord injury	None
CK	Female	57	Yes	Yes	Single	06	Grade 12	Mobility impairment – uses crutches	Yes. I need somebody to send wherever I want to go and buy some products
HS	Male	51	Yes	Yes	Single	07	Grade 09	Spinal cord injury	None
JM	Male	41	Yes	Yes	Single	02	Primary school	Spinal cord injury	Yes. Especially when it rains because I cannot move around or go out to people
MB	Male	43	Yes	Yes	Married	06	Grade 08	Spinal cord injury	None
PM	Male	42	Yes	Yes	Married	07	Primary school	Spinal cord injury	Yes. I cannot fully function
SN	Male	52	Yes	Yes	Single	07	Grade 11	Spinal cord injury	Yes. Being a wheelchair user and accessing transport
TM	Male	45	Yes	Yes	Single	04	None	Spinal cord injury	None
BN	Male	35	Yes	Yes	Single	03	Grade 11	Spinal cord injury	Yes. I cannot drive, but most of my work needs driving
GM	Male	63	Yes	Yes	Single	13	Primary school	Lower limbs bilateral amputee	Uncertain

### Themes

Two main themes and their respective categories and subcategories emerged from the data. Quotations with persons with disabilities’ initials, gender and age are used to support the findings from the semi-structured interviews. Table 2 depicts these two themes, namely, microenterprises and key role players involved in self-employment for persons with disabilities in a rural setting of KwaZulu-Natal.

**TABLE 2:** Themes, categories and subcategories of microenterprises and key role players involved in self-employment for persons with disabilities in a rural setting of KwaZulu–Natal.

Themes	Categories	Subcategories
1. Running microenterprises – self-initiated and maintained	1.1 Categories of the microenterprises – ‘There are many businesses’ which entail buying and selling, offering services or product production	1.1.1 Usage of hands and minds by persons with disabilities to engage in various microenterprises 1.1.2 Services and tangible products as part of categories of microenterprises
	1.2 The approach used by persons with disabilities to start self-employment – They ‘Start anything on a small scale’ to cut costs	1.2.1 The existence or non-existence of programmes for persons with disabilities to start microenterprises 1.2.2 The importance of microenterprise programmes and self-taught skills used by persons with disabilities in self-employment 1.2.3 Strategic approaches to self-employment: minimising expenses and scaling up gradually in microenterprises
	1.3 The reason persons with disabilities become self-employed – ‘If you are self-employed, it is different’ for example, it is a form of productivity that allows a sense of control	1.3.1 The feasibility and uniqueness of earning a living through self-employment 1.3.2 Self-employment as a form of productivity for persons with disabilities
2. Multiple key role players should be involved in self-employment	2.1 ‘We [*persons with disabilities*] must rise’ and get involved in self-employment individually and as a collective	2.1.1 Persons with disabilities value being proactive in self-employment 2.1.2 Unity and peer support as motivators for persons with disabilities in self-employment
	2.2 It would help persons with disabilities ‘Approaching people [*key role players*] for assistance’	2.2.1 The involvement of key role players in different settings 2.2.2 The roles of various key role players in supporting persons with disabilities in self-employment

### Theme one: Running microenterprises – Self-initiated and maintained

Theme one describes the occupation of self-employment in microenterprises owned by persons with disabilities under three categories as detailed in Table 1. These subcategories are discussed in detail.

#### Categories of the microenterprises – ‘There are many businesses’ which entail buying and selling, offering services or product production

**Usage of hands and minds by persons with disabilities to engage in various microenterprises:** As observable in their environmental and social contexts, persons with disabilities reported that more can be achieved in self-employment when one utilises their functional limbs following an injury or diagnosis. Refer to supporting quotes from persons with disabilities as follows:

‘… [*t*]here are many disabled people that engage in self-employment, as you can see my neighbours that I work with here. They also use their hands to work.’ (CK, Female, 57)

‘There is a lot that they [*persons with disabilities*] can do with their hands.’ (BN, Male, 35)

‘Businesses are there. There are many businesses that are owned by people with disabilities.’ (SN, Male, 52)

**Services and tangible products as part of categories of microenterprises:** Persons with disabilities indicated that they engage in self-employment by running businesses on a small scale, such as crafts, art, entertainment, repairs, farming and construction. Their microenterprises typically involve the production and sale of tangible products, which they either manufacture and sell or procure and resell to customers. Examples of these products include, but are not limited to, handicrafts, jewellery and brickmaking. Additionally, some of their microenterprises are skill-based or service-oriented, entailing the provision of services such as towing cars, repairs for shoes and electronics repairs, and entertainment services such as stage theatre.

#### The approach used by persons with disabilities to start self-employment – They ‘Start anything on a small scale’ to cut costs

**The existence or non-existence of programmes for persons with disabilities to start microenterprises:** Persons with disabilities shared their perspectives on available programmes and the importance of training related to self-employment. They reported that they are unaware of self-employment programmes for persons with disabilities. In addition, they emphasised the challenges they face in accessing the opportunities that could assist them in self-employment. Following are some supporting quotes from persons with disabilities:

‘No, I do not know anything about that [*programmes*]. This is new to me. So, I have never met anyone before [*talking about that*].’ (BN, Male, 35)

‘No, I am not aware … Sometimes we hear about opportunities [*related to microenterprises*], but the big problem is that we cannot access them. You find that we hear that there are opportunities for people with disabilities, but in the end, they do not reach us.’ (PM, Male, 42)

**The importance of microenterprise programmes and self-taught skills used by persons with disabilities in self-employment:** Despite lacking training, persons with disabilities reported that they are in favour of business training programmes. They mentioned that more persons with disabilities would be inclined to pursue entrepreneurial ventures if there were dedicated programmes to provide them with requisite support and guidance. The persons with disabilities indicated that training would enhance their business skills and knowledge, especially those without formal educational backgrounds. They further reported that training would ultimately contribute to improved customer satisfaction. Some persons with disabilities shared the following:

‘In fact, I think if there was a programme to begin with, everyone who is disabled would have started a company, knowing that there is that particular programme.’ (HS, Male, 51)

‘Most of us, especially the older generation, we did not get any formal education because of the circumstances [*apartheid in South Africa*] at that time.’ (TM, Male, 45)

‘You need training, you need to learn how to manage and treat people, how to make adverts and so on. All those things are needed. If you do not have those things, then nothing is going to work.’ (SN, Male, 52)

Persons with disabilities reported that they did not receive any training or other self-employment opportunities, largely because of inaccessible resources. They noted that they relied on self-directed learning or started self-employment ventures utilising their innate skills, planning, execution, trial and error, and observation of entrepreneurial peers. Some microenterprises required their involvement either physically or cognitively, or in a combination of both capacities. Persons with disabilities acknowledged that self-employment is not always easy and reported that they adopt a resilient mindset, avoid quitting and instead persevere, investing effort and demonstrating commitment to remain self-employed. Following are some supporting remarks from the persons with disabilities:

‘I started teaching myself at home, sewing a few things … I then expanded my knowledge by doing the work.’ (CK, Female, 57)

‘You find that it [*self-employment*] is difficult even if you are not disabled … It is not like I was presented with an opportunity. I thought hard about it and worked hard to start … There is nothing that says I have to be involved physically in the business all the time.’ (PM, Male, 42)

‘I use my hands for some things. I use my brain for some things … You also have to pursue and follow-up on your work once you have started it. You have to keep working on it until you succeed.’ (SN, Male, 52)

**Strategic approaches to self-employment: Minimising expenses and scaling up gradually in microenterprises:** Capital and transport were reported as constrained resources that have adverse effects when persons with disabilities initiated their microenterprises, as these are crucial for sustaining day-to-day operations and facilitating business growth. The persons with disabilities problem-solved by saving from their government disability grants, adopting a phased approach to business development by beginning small and scaling and leveraging their home environment by operating rent-free. They also utilised informal marketing strategies, such as word of mouth, to raise awareness among prospective customers about their businesses and the products or services they offer. Some persons with disabilities shared the following:

‘[*To cut costs*] I work from home. People come to me for my services.’ (JM, Male, 41)

‘You can start anything on a small scale, but you will eventually reach the level where you are fully self-employed.’ (MB, Male, 43)

‘[*Regarding business growth*] things later changed, and now I have people that work for me.’ (BN, Male, 35)

‘If you want to reach a certain place at a certain time … Transportation is difficult when you are disabled.’ (TM, Male, 45)

#### The reason persons with disabilities become self-employed – ‘If you are self-employed, it is different’ for example, it is a form of productivity that allows a sense of control

**The feasibility and uniqueness of earning a living through self-employment:** Persons with disabilities provided a rationale for their involvement in self-employment and associated benefits. Key factors such as the scarcity of employment opportunities in the open labour market, particularly for persons with disabilities, led to their exploration of self-employment in microenterprises. The benefits highlighted were predominantly at the individual level encompassing financial gains, a sense of control, ownership and self-growth or personal development. Following are supporting quotes from some persons with disabilities:

‘If you are self-employed, it is different to when you are employed by someone else … [*It is a way to*] earn their [*persons with disabilities*] own money by working with their hands … I know that when people call, they call me.’ (BN, Male, 35)

‘[*As an owner*] it is one way that is almost certain that nothing will disturb your work … I no longer run out of money to buy bread … For us [*persons with disabilities*] to develop, it is best that we engage in self-employment.’ (MB, Male, 43)

‘… [*i*]t is not easy for us to find employment. [*Self-employment is for*] a person that loves doing what they are doing [*to*] earn a living.’ (CK, Female, 57)

‘[*I am*] trying to earn a living in business based on my own knowledge.’ (TM, Male, 45)

Persons with disabilities also reported that their self-employment earnings made a contribution to their households, enabling them to earn a sustainable living and positively impact the well-being of their loved ones. Some persons with disabilities stated:

‘We [*persons with disabilities*] do try to engage in self-employment because of the circumstances of life. If you have a family, you have to provide for them.’ (PM, Male, 42)

‘[*I am self-employed to*] make the situation better at home, and life to be better at home, for my family.’ (BN, Male, 35)

**Self-employment as a form of productivity for persons with disabilities:** Persons with disabilities reported that they are motivated and place value in productivity. They believe that although formal work opportunities are generally limited for persons with disabilities, they can still maintain productivity and autonomy by exploring self-employment as an alternative to relying solely on government social initiatives such as disability grants. Persons with disabilities stated:

‘Actually, we [*as persons with disabilities*] do not even want that [*disability*] grant. We take it because there is nothing that we can do. We want jobs. We want to be self-employed.’ (SN, Male, 52)

‘[*I engage in self-employment to avoid*] just staying at home and sleeping the whole time.’ (MB, Male, 43)

### Theme two: Multiple key role players should be involved in self-employment

Theme two describes persons with disabilities’ perspective regarding the key role players involved in facilitating their engagement in self-employment included a crucial role played by authority figures and other persons with disabilities. Their responses are summarised below.

#### ‘We [*persons with disabilities*] must rise’ and get involved in self-employment individually and as a collective

**Persons with disabilities value being proactive in self-employment:** Persons with disabilities perceive themselves as proactive agents in finding and engaging in self-employment thereby enhancing the sustainability of their microenterprises, such fostering customers satisfaction. In addition to deriving personal fulfilment from being self-employed, persons with disabilities acknowledge the importance of self-reliance and proactivity by, for example, pursuing opportunities such as training and business leads. Following are some supporting comments:

‘We [*person with disabilities*] must rise. We must not sit in corners …’ (CK, Female, 57)

‘[*Person with disabilities must*] go out to seek assistance.’ (JM, Male, 41)

‘[*I must*] go to look for opportunities as someone that owns a business … If ever I hear about opportunities, I go there.’ (HS, Male, 51)

‘We [*persons with disabilities*] want to be a part of everything that is happening so that we can be visible in the community and to the people that occupy senior positions so that they can see that we also need help at all times, just like all other people.’ (MB, Male, 43)

Persons with disabilities also emphasised the significance of respecting their customers – one of their crucial responsibilities in self-employment being developing effective customer service skills. Dedication, putting in the work and delivering high-quality products and services were reported by persons with disabilities as essential for success. Following are some supporting comments from persons with disabilities:

‘I can say it is important that when I do my work and do it well, maybe it can encourage another person to not just sit idly.’ (TM, Male, 45)

‘[*Other persons with disabilities*] are stressed out and are giving up because things are not going the way they want [*but they*] must not think just because they are disabled, it means they must produce sub-standard quality of work.’ (BN, Male, 35)

**Unity and peer support as motivators for persons with disabilities in self-employment:** Beyond individual responsibility, mutual support and collective responsibility were emphasised as essential. For instance, they noted it is vital to serve as positive role models and motivate one another, particularly those experiencing challenges in their business or personal lives. Persons with disabilities, as key role players, acknowledge the benefits of collaboration and collective action. They also advocate for peer support and uplifting those in need the most. People with disabilities recognise the value of collective strength, and some have initiated collaborative endeavours. For instance, as a collective, they have approached the government and private sector for assistance in their microenterprises. Here are some quotes from persons with disabilities:

‘We should help others [*persons with disabilities*] and explain to them that they should not just sit and expect pity because they are disabled. No! They must also get up and try to do what they can to be able to earn a living. So, we must motivate each other and not be selfish. We should help others. When you see that the other person has a challenge, give them a chance as well.’ (CK, Female, 57)

‘Those of us [*persons with disabilities*] who are ahead would have to visit others who are not exposed and tell them about life, what happens when you go out there, and what happens when you stay at home. So, when we tell people about our life experiences, they will realise that we are advising.’ (GM, Male, 63)

‘What can make us recognisable is to establish our own thing [*work collectively or as a unit*].’ (TM, Male, 45)

‘We registered companies … right now, we have formed a cooperation or joint venture … We have registered it with the municipality … We are going to seek funding in different departments, including the Department of Transport and municipalities and elsewhere … We intend approaching everyone [*potential funders*], including Coca-Cola, Lotto and so on.’ (HS, Male, 51)

#### It would help persons with disabilities ‘Approaching people [*key role players*] for assistance’

**The involvement of various key role players in different settings:** Persons with disabilities reported that the key role players who should assist them in finding and maintaining self-employment include government agencies and municipalities, hospital multidisciplinary teams (such as doctors, occupational therapists and physiotherapists) and private institutions. Assistance could be with training and funding initiatives. The person with disabilities reported that the government should not only incentivise their businesses given that the administrative processes for running a business are costly, such as company yearly fees, but also cut the red tape or streamline bureaucratic processes. They advocated for simplifying the tender bidding process, rather than rejecting their bids because of perceived documents deficiencies. Regarding projects, mainly local or in their area, persons with disabilities felt that those in position of authority should uphold their rights by allocating them the prescribed percentage of work, as per South Africa’s procurement law. Following are supporting comments from some persons with disabilities:

‘I think the government or those in authority in government, whose job is to look after people with disabilities, are the ones that should fight for our [*persons with disabilities’*] rights.’ (HS, Male, 51)

‘The [*government*] departments also put age restrictions and tell you about 35 years and so on. All of those things restrict us [*persons with disabilities*] … When you try to do this, they [*government officials*] will tell you that something is missing. So, these are the things that make us to end up not succeeding.’ (SN, Male, 52)

‘Yes, the municipality helps us sometimes or provides sponsorship when there are sporting events. They do help us sometimes.’ (BN, Male, 35)

‘There are many [*business related*] things that are needed [*persons with disabilities could use key role players’ help*].’ (CK, Female, 57)

**The roles of various key role players in supporting persons with disabilities in self-employment:** Persons with disabilities reported that beyond assisting with documentation to confirm their disability, hospital staff offer holistic support, including emotional and psychosocial support, such as encouraging them to reintegrate into their lives following an injury or diagnosis. Other support mechanisms reported by persons with disabilities are more informal in nature, existing at an individual than a programme level. These initiatives, facilitated by hospital staff, entail assisting persons with disabilities to access business infrastructure, such as a building, and navigate further support services, including business registration. Some persons with disabilities had this to say:

‘… [*w*]e [*persons with disabilities*] were assisted by the people from the hospital. They found a place for people with disabilities that have skills to work from and be able to earn a living … So, they helped us by getting a person to teach us so I [*we*] could develop the little knowledge that I [*we*] had.’ (CK, Female, 57)

‘They [*key role players*] play a role in ensuring that they help us [*persons with disabilities*] or guide us to platforms where we can get help.’ (MB, Male, 43)

‘[*Professionals advise us*] not to stay at home because staying at home would mean that I just sleep and wake up and sit on the wheelchair.’ (JM, Male, 41)

## Discussion

The study explored persons with disabilities’ experiences in initiating and maintaining self-employment in microenterprises and their perceptions of key role players in self-employment in microenterprises. The demographics will be discussed first.

### Demographics

The participants had physical disability and limited literacy levels. They shared similar demographics with those reported in the literature (Monareng et al. 2021; Shakespeare et al. 2019; Viriri & Makurumidze 2014; Wasike & Likoko 2013) as their education level ranged from no education to grade 11. All participants were adults of working age between the ages of 35 and 65, mostly males. These findings align with those reported in other research (Gamieldien & Van Niekerk 2017; Monareng et al. 2021; Wasike & Likoko 2013). Furthermore, persons with disabilities in this research had an average of six dependents. These findings are corroborated by a study conducted by Waskike et al. (2013), where the participants’ average number of dependents was less than 10. The participants were single and sole owners of businesses with an average of 5 years of self-employment (Alcock 2018). This period exceeds the 3-year survival threshold, during which start-up businesses will likely fail (Alcock 2018). Overall, these findings confirm that persons with disabilities in this research successfully operated microenterprises.

### Persons with disabilities’ experiences in initiating and maintaining self-employment in microenterprises

Persons with disabilities in the rural setting of KwaZulu-Natal leverage available resources to engage in self-employment in microenterprises (American Occupational Therapy Association 2020), which provides them with a livelihood. These findings align with Pagán’s (2009) findings on self-employment being a preference or an alternative over conventional work among persons with disabilities (Gamieldien & Van Niekerk 2017). Regarding the types of microenterprises, findings in this research are consistent with those found in other parts of South Africa (Charman et al. 2017; Gamieldien & Van Niekerk 2017; Maziriri, Madinga & Lose 2017; Rogan & Skinner 2018; Valodia 2007) and mirrored in other African countries such as, but not limited to, Kenya, Malaysia, Sierra Leone and Tanzania (Binti et al. 2012; Daniel 2019; Shakespeare et al. 2019; Witchger Hansen & Blaskowitz 2018) where products are sold or services are rendered. These types of businesses exist in ordinary community settings and are significant in that they address community daily needs.

Persons with disabilities utilise their remaining functions, such as functional upper limbs (to produce products) and executive cognitive function (for planning and problem-solving), to engage in and perform business tasks so that they can earn and provide for their families (Gamieldien & Van Niekerk 2017; Morwane et al. 2021). The limited resources in contexts such as rural areas do not permit the traditional business set-up procedures (Blanck et al. 1999; Isaacs et al. 2007; Wasike & Likoko 2013). For instance, formal business training, capital and support are essential components of business, but they are not accessible to persons with disabilities in the rural setting of KwaZulu-Natal. These are similar challenges faced by those operating businesses in low-resourced urban settings such as Sebokeng Township in Gauteng, South Africa (Charman et al. 2017; Maziriri et al. 2017; Wasike & Likoko 2013). Consequently, persons with disabilities overcome and adapt by starting where they are with what they have (Daniel 2019; Shakespeare et al. 2019). They cut costs related to their operations, such as starting at home without rent or not purchasing a business site (Lorenzo, Van Niekerk & Mdlokolo 2007). Such initiatives enable them to conserve resources and avoid travel and daily set-up costs, synonymous with a microenterprise without a permanent business site (Charman et al. 2017; Daniel 2019; Gamieldien & Van Niekerk 2017). While some participants received informal training or apprenticeship from a relative or friend, some were self-taught entrepreneurs who relied on trial and error and continuous problem-solving (Wasike & Likoko 2013). These findings highlight how feasible it is for persons with disabilities to engage in self-employment despite challenges and insufficient resources.

The small sizes of these microenterprises require less capital or funding to start (Yu & Roos 2018), that is, fewer barrier to entry (Mahadea & Khumalo 2020). Such funding could be raised from close social networks, such as friends and family (Mersland 2005), who in turn support the business in the form of labour or becoming customers (Gamieldien & Van Niekerk 2017; Van Niekerk, Lorenzo & Mdlokolo 2006). On the other hand, some microenterprises may be self-funded using a government disability grant (South African Government 2023). The usage of disability grants in this instance is contrary to the study conducted by Engelbrecht and Lorenzo (2010), which indicated that a disability grant may perpetuate disempowerment in cases where the recipients are passive or show no interest in being productive.

As such, given the complexity of challenges associated with self-employment, the nature of these businesses requires sustained patience and commitment (Isaacs et al. 2007; Lorenzo et al. 2007). This may imply that benefits, such as earnings, may not always be immediately tangible or consistent, as they can only be fully realised later.

There are benefits beyond starting the microenterprises out of necessity, which may entail, but are not limited to, enhanced self-subsystem or efficacy, such as motivating persons with disabilities to aspire to and lead a positive, meaningful life (Soeker, Abbas & Karachi 2023). Other benefits for persons with disabilities who are self-employed include becoming active community members and having the ability to provide (Gamieldien & Van Niekerk 2017; Shakespeare et al. 2019). These benefits align with the fulfilment of key health determinants and contribute towards living above the poverty line (Gamieldien & Van Niekerk 2017; Naledi, Barron & Schneider 2011). Other benefits, which are not the primary motive for such businesses, are securing a permanent business location (Lorenzo et al. 2007) and expanding their operations through hiring (Gamieldien & Van Niekerk 2017; Shakespeare et al. 2019), that is growing the business. Despite persons with disabilities’ reasons for getting into self-employment, they need targeted training and support for adequate structures and systems that enable sustainability (Chimucheka 2014; Isaacs et al. 2007; Wasike & Likoko 2013).

### Persons with disabilities’ perceptions of key role players in self-employment in microenterprises

According to the persons with disabilities in this study, the responsibilities and roles of persons with disabilities in self-employment are not only towards themselves but also towards their customers and fellow persons with disabilities (Shakespeare et al. 2019). They must be proactive in upskilling themselves to advance business operations and leverage opportunities in their communities (Maseko et al. 2023), such as at the municipality level. Moreover, the participants in this study held themselves to open-labour market standards and espoused the need to be dedicated and committed (Shakespeare et al. 2019), and to use a customer-oriented approach (Gamieldien & Van Niekerk 2017) as well as not use their disability as an excuse for offering inferior products or services.

Supported by Shakespeare et al. (2019), persons with disabilities perceive role modelling by peers who are ahead in microenterprises, as essential. Furthermore, persons with disabilities often operate more in isolation, although they perceive networking and information sharing as crucial (Maziriri et al. 2017; Viriri & Makurumidze 2014). They believe supporting each other will contribute towards sustaining their businesses. Despite lacking the necessary resources, one would conclude that they perceive themselves as a homogenous group with shared interests and needs, hence the act of collegiality. One participant stated: ‘We [*persons with disabilities*] must rise’ (CK, Female, 57), emphasising togetherness.

Although persons with disabilities, in this study, reported on the importance of receiving assistance with self-employment in microenterprises in and outside the hospital setting (Morwane et al. 2021; Van Biljon et al. 2016), the aid was found to be insufficient. In a hospital setting, vocational rehabilitation in South Africa typically occurs after medical intervention or when suitable persons with disabilities are medically stable (Pefile, Mothabeng & Naidoo 2016; Van Niekerk n.d.). Work placement options, such as self-employment (Chimara et al. 2022; Gamieldien & Van Niekerk 2017), should ideally be initiated during the holistic rehabilitation phase before persons with disabilities leave the hospital. A professional in the community should carry over this service as part of primary healthcare re-engineering (Naledi et al. 2011). Similarly, Chimara et al. (2022) and Maseko et al. (2023) stated that for sustainability, an intervention must extend beyond the hospital setting, especially in low-resourced countries or settings.

Outside the hospital setting, leading professionals and community key role players, such as the government, private, business or non-government organisations, should collaborate to ensure continuity of intervention and sustainability of services. The key role players can do such by offering necessary services to persons with disabilities, such as psychosocial support, physical rehabilitation and business income generation projects (Lorenzo et al. 2007; Mahadea & Khumalo 2020; Parker 2012; Soeker 2017). The government should facilitate a conducive self-employment environment at the legislative level (Mahadea & Khumalo 2020; Pefile et al. 2016).

Maseko et al. (2023) found that initiatives based in the community are beneficial as they reduce costs and are effective if run well. Furthermore, they recommended that for effective, sustainable interdisciplinary community-based intervention and collaboration, the following models should be incorporated: shared care model, self-management model and community-based rehabilitation model. Coordinating such initiatives and implementing such models would benefit persons with disabilities, including those in self-employment, such as those in this research.

## Conclusion

In the rural context of KwaZulu-Natal, the findings from this study indicate that persons with disabilities engage in self-employment in microenterprises, which they self-initiate and maintain or sustain. This research also provides insight into how persons with disabilities start their microenterprises and their rationale for pursuing self-employment. Although a challenging and demanding field, persons with disabilities demonstrated proactivity and resilience in overcoming associated challenges such as limited access to resources. The benefits of self-employment were evident, including reintegrating persons with disabilities into their communities and enabling them to provide for their families.

The findings also revealed that self-employment roles and responsibilities of persons with disabilities and key role players based in and outside the hospital setting are essential in facilitating successful self-employment outcomes. This includes providing assistance with psychosocial aspects and referring further where necessary. Moreover, community resources must be mobilised and leveraged to support persons with disabilities in self-employment in microenterprises after discharge from the primary admission institution. Ultimately, this study’s findings should inform those involved to better understand this occupation and effectively mobilise community resources to support persons with disabilities.

### Implications and recommendations

The implications and recommendations of this research are:

- The limited availability of recent research on persons with disabilities in self-employment presents a notable knowledge gap. To address this shortfall, future studies should focus on producing timely, diverse and context-specific data through engagement with key stakeholders, such as service providers, disability advocacy groups and policymakers.
- Key role players in self-employment should foster an enabling environment that encourages, mobilises and supports persons with disabilities. Peer support networks could be facilitated through support groups among persons with disabilities who have similar interests and needs in the community.
- A comprehensive directory of successful or profitable self-employment microenterprises must be compiled, particularly in resource-constrained areas.
- To gain a more nuanced understanding of this field, future research should undertake a comprehensive analysis of microenterprises, including, but not limited to, compiling a list and providing a detailed examination of their operational structures and processes.
- Colleagues involved in vocational rehabilitation are encouraged to consider including findings from this research in their practice and curriculum (for those in academia and special needs schools, respectively). Those involved should find, capacitate and explore innovative strategies to enhance their services beyond the hospital setting and incorporate self-employment in microenterprises for persons with disabilities.
- The government should develop targeted strategies around disability grants and incentivise self-employment initiatives by persons with disabilities. A policy brief on this subject should be considered and directed to the government.

### Strengths and limitations

#### Strengths

This research adds knowledge to the limited field of self-employment, specifically for persons with disabilities in an African context.

#### Limitations

Although not an explicit exclusion or inclusion criterion, this study could only reach persons with physical disabilities. Future research should consider participants with other disabilities, for example, mental illness. Although the findings are valuable, generalisation may be challenging, given the small sample size, which is synonymous with qualitative studies.
